# 50. Pilot Study on Offering HIV Pre-Exposure Prophylaxis (PrEP) to People Who Inject Drugs (PWID) in the Inpatient Setting

**DOI:** 10.1093/ofid/ofab466.050

**Published:** 2021-12-04

**Authors:** Terry A Marryshow, Jose Caro

**Affiliations:** Tufts Medical Center

## Abstract

**Background:**

Due to the ongoing opioid epidemic, PWID represent an increasingly high-risk population for HIV infections in the United States, accounting for 10% of all new HIV diagnoses in 2018 and 12.5% of all deaths among people living with HIV. PrEP is an effective means of preventing HIV, though uptake has been low among PWID, possibly due to low access to care. Inpatient admissions may represent missed opportunities for provision of PrEP to PWID.

**Methods:**

Inpatient prescriptions for tenofovir disoproxil fumarate-emtricitabine (TDF-FTC) from 10/2019 to 8/2020 were analyzed to assess baseline provision of PrEP to PWID. Physicians on the Infectious Diseases ward service were anonymously queried on perceived barriers and their practices regarding provision of PrEP to PWID. PWID admitted from 9/2020 to 5/2021 were approached at bedside, provided counseling on PrEP and offered initiation prior to discharge. We analyzed patient perceptions and acceptance of PrEP.

**Results:**

16 total prescriptions for TDF-FTC were provided at discharge from 10/2019 to 8/2020, with 0 being for PrEP in PWID. The 8 physicians surveyed estimated caring for an average 4 PWID per week of service. 5/8 physicians reported that at least one PWID was offered PrEP during their most recent week of service. The most commonly reported physician barrier to prescribing PrEP was uncertainty regarding adherence and follow up (5/8). 30 patients were approached, with 14 reporting prior knowledge of PrEP. 18 were willing to engage in further education. Only 4 were accepting of PrEP, with 2 provided prescriptions. Of those declining, 13 denied equipment sharing, 4 denied active drug use, 7 stated a commitment to future abstinence, 3 were unwilling to adhere to a daily medication, 2 declined due to concerns of adverse effects and 1 due to concerns regarding stigma.

Table 1. Physician Reported Barriers to Prescribing PrEP (n = 8)

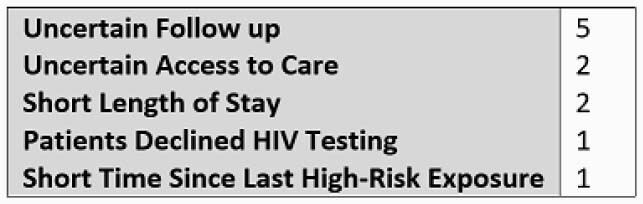

Table 2. Patient Knowledge and Acceptance of PrEP (n = 30)

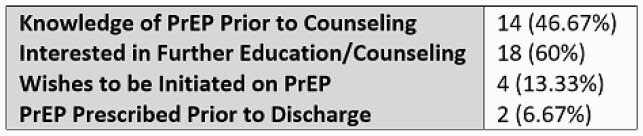

Table 3. Patient Reasons for Declining PrEP (n = 30)

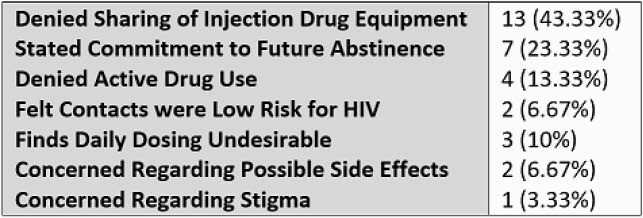

**Conclusion:**

In this pilot study, few admitted PWID were accepting of PrEP. Attempts to initiate PrEP in PWID in the inpatient setting may not be effective at our institution. The most common reason for declining was low self-perceived risk of HIV acquisition; however, a significant proportion of patients showed interest in further education. Therefore, the inpatient setting may be a valuable site of initial counseling and referral for future potential provision of PrEP in the outpatient setting.

**Disclosures:**

**All Authors**: No reported disclosures

